# Action perception as hypothesis testing

**DOI:** 10.1016/j.cortex.2017.01.016

**Published:** 2017-04

**Authors:** Francesco Donnarumma, Marcello Costantini, Ettore Ambrosini, Karl Friston, Giovanni Pezzulo

**Affiliations:** a Institute of Cognitive Sciences and Technologies, National Research Council, Rome, Italy; b Centre for Brain Science, Department of Psychology, University of Essex, Colchester, UK; c Laboratory of Neuropsychology and Cognitive Neuroscience, Department of Neuroscience and Imaging, University G. d'Annunzio, Chieti, Italy; d Institute for Advanced Biomedical Technologies – ITAB, Foundation University G. d'Annunzio, Chieti, Italy; e Department of Neuroscience, University of Padua, Padua, Italy; f The Wellcome Trust Centre for Neuroimaging, UCL, London, UK

**Keywords:** Active inference, Action observation, Hypothesis testing, Active perception, Motor prediction

## Abstract

We present a novel computational model that describes action perception as an *active* inferential process that combines *motor prediction* (the reuse of our own motor system to predict perceived movements) and *hypothesis testing* (the use of eye movements to disambiguate amongst hypotheses). The system uses a generative model of how (arm and hand) actions are performed to generate hypothesis-specific visual predictions, and directs saccades to the most informative places of the visual scene to test these predictions – and underlying hypotheses. We test the model using eye movement data from a human action observation study. In both the human study and our model, saccades are proactive whenever context affords accurate action prediction; but uncertainty induces a more reactive gaze strategy, via tracking the observed movements. Our model offers a novel perspective on action observation that highlights its *active* nature based on prediction dynamics and hypothesis testing.

## 1 Introduction

The ability to recognize the actions of others and understand their underlying intentions is essential for adaptive success in social environments – and we humans excel in this ability. It has long been known that brain areas such as superior temporal sulcus (STS) are particularly sensitive to the kinematic and dynamical signatures of biological movement that permit its fast recognition (Giese and Poggio, 2003, Puce and Perrett, 2003). However, the neuronal and computational mechanisms linking the visual analysis of movement kinematics and the recognition of the underlying action goals are more contentious.

In principle, the recognition of action goals might be implemented in perceptual and associative brain areas, similar to the way other events such as visual scenes are (believed to be) recognized, predicted and understood semantically. However, two decades of research on action perception and mirror neurons have shown that parts of the motor system deputed to specific actions are also selectively active during the observation of the same actions when others perform them. Based on this body of evidence, several researchers have proposed that the motor system might support – partially or totally – action understanding and other functions in social cognition (Kilner and Lemon, 2013, Rizzolatti and Craighero, 2004). Some theories propose an automatic mechanism of *motor resonance*, according to which the action goals of the performer are “mirrored” in the motor system of the perceiver, thus permitting an automatic understanding (Rizzolatti, Fadiga, Gallese, & Fogassi, 1996). Other theories highlight the importance of (motor) prediction and the covert reuse of our own motor repertoire and internal models in this process. For example, one influential proposal is that STS, premotor and parietal areas are arranged hierarchically (in a so-called *predictive coding* architectural scheme) and form an *internal generative model* that predicts action patterns (at the lowest hierarchical level) as well as understanding action goals (at the higher hierarchical level). These hierarchical processes interact continuously through reciprocal top-down and bottom-up exchanges between hierarchical levels, so that action understanding can be variously influenced by action dynamics as well as various forms of prior knowledge; such as the context in which the action occurs (Friston et al., 2011, Kilner et al., 2007). Numerous other theories point to the importance of different mechanisms besides mirroring and motor prediction, such as Hebbian plasticity or visual recognition (Fleischer et al., 2013, Heyes, 2010, Keysers and Perrett, 2004), see Giese and Rizzolatti (2015) for a recent review. However, these theories implicitly or explicitly consider action observation as a rather passive task, disregarding its enactive aspects, such as the role of *active information sampling* and *proactive eye movements*.

In everyday activities involving goal-directed arm movements, perception is an active and not a passive task (Ahissar and Assa, 2016, Bajcsy et al., 2016, O'Regan and Noe, 2001); and eye movements are proactive, foraging for information required in the near future. Indeed, eyes typically shift toward objects that will be eventually acted upon, while being rarely attracted to action irrelevant objects (Land, 2006, Land et al., 1999, Rothkopf et al., 2007). A seminal study (Flanagan & Johansson, 2003) showed that when people observe object-related manual actions (e.g., block-stacking actions), the coordination between their gaze and the actor's hand is very similar to the gaze-hand coordination when they perform those actions themselves. In both cases, people proactively shift their gaze to the target sites, thus anticipating the outcome of the actions. These findings suggest that oculomotor plans that support action performance can be reused for action observation (Flanagan & Johansson, 2003) and might also support learning and causal understanding of these tasks (Gredebäck and Falck-Ytter, 2015, Sailer et al., 2005).

Here we describe and test a novel computational model of action understanding and accompanying eye movements. The model elaborates the predictive coding framework of action observation (Friston et al., 2011, Kilner et al., 2007) but significantly extends it by considering the specific role of active information sampling. The model incorporates two main hypotheses. First, while most studies implicitly describe action observation as a passive task, we cast it as an active, *hypothesis testing* process that uses a generative model of how different actions are performed to generate hypothesis-specific predictions, and directs saccades to the most informative (i.e., salient) parts of the visual scene – in order to test these predictions and in turn disambiguate among the competing hypotheses (Friston, Adams, Perrinet, & Breakspear, 2012). Second, the generative model that drives oculomotor plans across action performance and observation is the same, which implies that the motor system drives predictive eye movements in ways that are coherent with the unfolding of goal-directed action plans (Costantini et al., 2014, Elsner et al., 2013).

We tested our computational model against human data on eye movement dynamics during an action observation task (Ambrosini, Costantini, & Sinigaglia, 2011). In the action observation study, participants' eye movements were recorded while they viewed videos of an actor performing an unpredictable goal-directed hand movement toward one of two objects (targets) mandating two different kinds of grip (i.e., a small object requiring a precision grip or a big object requiring a power grasp). To counterbalance the hand trajectories and ensure hand position was not informative about the actor's goal, actions were recorded from the side using four different target layouts. Before the hand movement, lasting 1000 msec, the videos showed the actor's hand resting on a table (immediately in front of his torso) with a fixation cross superimposed on the hand (1000 msec). Participants were asked to fixate the cross and to simply watch the videos without further instructions. In half of the videos, the actor preformed a reach-to-grasp action during which the preshaping of the hand (either a precision or a power grasp, depending on the target) was clearly visible as soon as the movement started (preshape condition), whereas in the remaining half, the actor merely reached for – and touched – one of the objects with a closed fist; that is, without preshaping his hand according to the target features (no shape condition). Therefore, there were four movement types, corresponding to the four conditions of a two factor design (pre-shape and target size); namely, no shape–big target, no shape–small target, pre-shape–big target and pre-shape–small target. The four conditions were presented in random order so that the actor's movement and goal could not be anticipated. The main result of this study was that participants' gaze proactively reached the target object significantly earlier when motor cues (i.e., the preshaping hand) were available. In what follows, we offer a formal explanation of this anticipatory visual foraging in terms of active inference.

## 2 Methods

Our computational model uses gaze and *active salient information sampling* to resolve uncertainty about the action being observed (Friston et al., 2012); i.e., a power grasp to a big object or a precision grip to a small object. The basic idea behind active information sampling rests on resolving uncertainty about the causes of sensations: namely, the action (cause) that explains the observed movements (sensations). In this setting, salience scores the information gain (or resolution of uncertainty) afforded by sampling information from a particular domain; here, a location in the visual field. To evaluate the salience (or epistemic value) of a putative saccade, it is necessary to predict what will be sampled from that location. In the active inference framework, predictions derive from *internal generative models* that essentially encode the probabilistic relations between causes (actions) and sensations (hand movements). Given a particular hypothesis (e.g., an actor reaching for a big object), the generative model can then predict the consequence of a saccade to a particular location (e.g., that a hand should be configured in power grasp). The resulting information gain, as measured by the reduction in posterior uncertainty under the expected outcome, then specifies the salience or epistemic value of the saccade – as a saccade to the hand location can test the predictions generated under the competing hypotheses (e.g., seeing the hand is configured in a power grip provides evidence for the hypothesis that the actor is reaching for a big object).

In our simulations, we evaluate the salience (*epistemic value*) of sampling every visual location under two competing hypotheses (the actor reaching for a big or a small object) and then weight the ensuing saliency maps by the posterior probability of each hypothesis. This corresponds to a Bayesian model average of salience maps over hypotheses (Penny, Mattout, & Trujillo-Barreto, 2006). Crucially, in the action observation setup considered here, this is an on-going process, because each new sensory sample changes posterior beliefs and therefore changes the (Bayesian model average) saliency map. Action observation is thus a process that unfolds in time, guided by active sampling of information that is most relevant (salient) to adjudicate among competing hypotheses.

Note that this definition of salience goes beyond (local) aspects of the visual input to consider goal-related information. Usually, salience is defined on the basis of visual features. In contrast, in active inference, salience is an attribute of a putative action; for example, where to look next. In this setting, salience is defined as the information gain based upon the expected resolution of uncertainty about explanations for sensory input. Mathematically, this *epistemic value* is equivalent to the mutual information between the hidden causes (explanations) and their sensory consequences, expected under a particular action (Friston et al., 2015). In this sense, salience is only defined in relation to active sampling of the environment, because it is a function of sensory samples conditioned upon an action. In our context, salience is brought further into the embodied or enactivist realm. This is because the hypotheses that need to be resolved through epistemic foraging are themselves contingent upon another's action. In the context of the action observation paradigm studied above – unlike other visual search tasks – the task requires an understanding of the action *goal* (e.g., ‘grasp the big object’) – as opposed to just predicting a sequence of video frames. The intentionality inherent in this task can be inferred by engaging the same oculomotor plans (and associated generative models) that support the execution of one's own goal-directed actions; e.g., the plan to fixate and grasp a big object (Flanagan & Johansson, 2003). The implicit generative or forward models influence what is salient and what is not salient in the visual scene. During action performance, the target location is salient because it affords goal-directed action. Reusing oculomotor plans for action observation thus explains why the target location becomes salient when it is recognized as the goal of the action – even before the performer's hand reaches it. However, there is an important difference between using oculomotor plans during action execution and observation. During action execution, we know the goal (e.g., big or small target). Hence, we know the target location and can saccade directly to it, without looking at our own hand. Conversely, during action observation, we need to infer which target the actor has in mind (e.g., the actor is reaching for the big target). To resolve uncertainty about which target to look at, we can first look at the actor's hand to see whether it is configured to pick up a small or large target. This means that the most salient location in the visual field changes as sensory evidence becomes available (as disclosed by the hand configuration and trajectory) – and subsequent changes in the observer's beliefs or hypotheses. Crucially, one would predict anticipatory saccade to the target object when, and only when, the actor's intentions or goal are disclosed by the hand configuration.

In summary, if the agent is confident about the goal, it should look at the target. However, if the agent is uncertain about the goal, it first needs to execute *epistemic actions* (i.e., collect evidence by looking at the actor's hand). This suggests that the salience of different locations (hand or objects) changes dynamically as a function of the agent's beliefs – a phenomenon that has been observed empirically (see above) and that we reproduce using simulations of active inference.

**Fig. 1:**
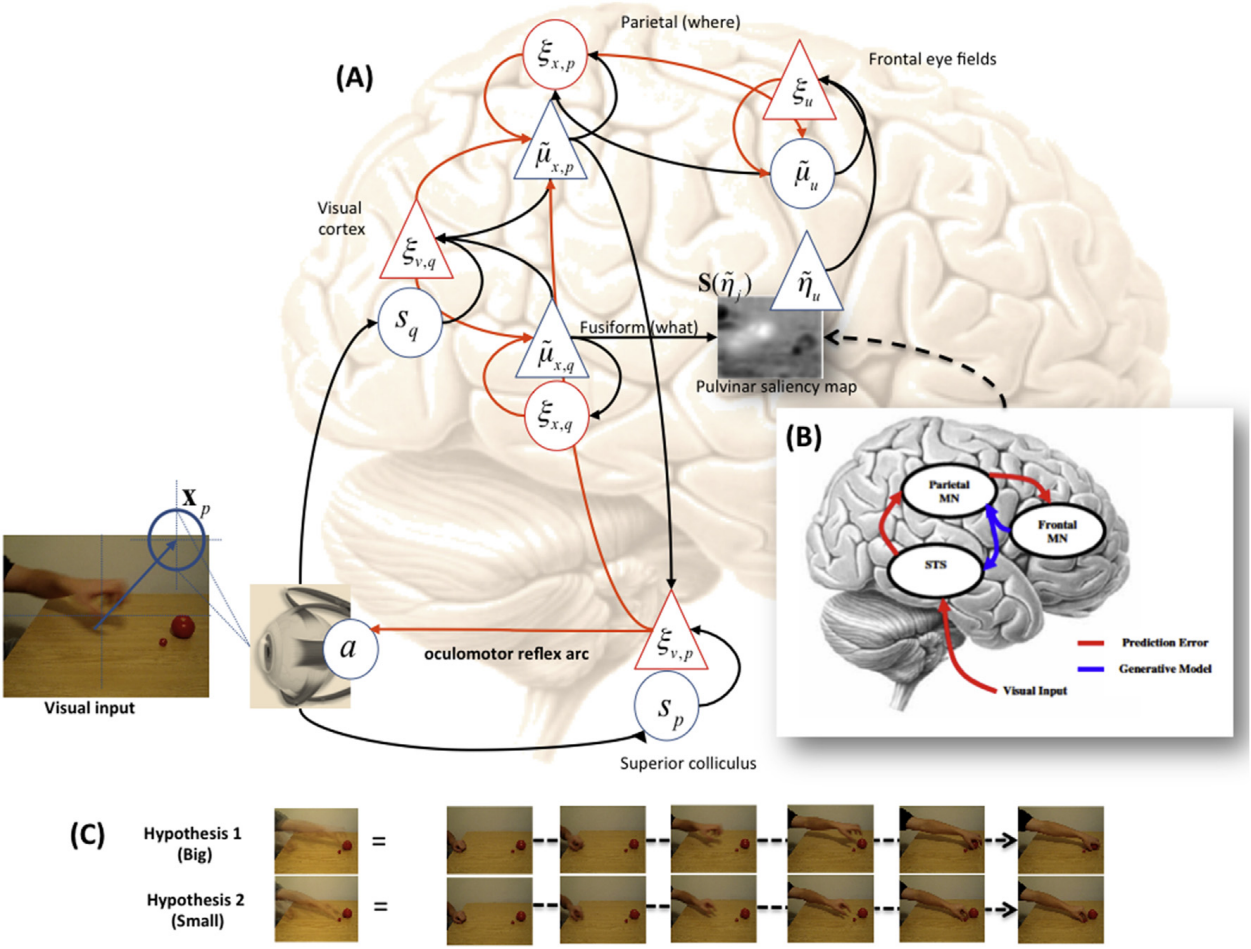
**Scheme of the computational model adopted in the study**. The system implicitly encodes a (probabilistic) model of which visual stimuli should be expected under the different perceptual hypotheses (e.g., if the action target is the big object, when doing a saccade to the next hand position I should see a power grasp) and uses the saccades to check if these expectations are correct – and in turn to revise the probability of the two hypotheses. Details of the procedure can be found in the main text and in Friston et al. (2012). (B) The pulvinar saliency map receives as input the (expected) position of task-relevant variables (e.g., expected hand position, to-be-grasped objects), weighted by their saliency, which in turn depends on the probability of the two competing hypotheses. Neurophysiologically, we assume that a hierarchically organized “action observation” brain network computes both the expected hand position (at lower hierarchical levels) and the probability of the two competing hypotheses (at higher hierarchical levels). The inset shows a schematic of the functioning of the action observation network according to predictive coding (Kilner et al., 2007). Here, action observation depends on reciprocal message passing between areas that lie lower in the predictive coding hierarchy (STS) and areas higher areas (parietal and prefrontal). The functioning of the action observation network is abstracted here using a Bayesian model (Dindo et al., 2011), see the Methods section for details. (C) This panel represents graphically the two competing hypotheses that are considered here. Note that here the hypotheses are not (only) about final states (hand on big *vs* small object) but describe also how the action will unfold in time: they correspond to sequences of (superimposed) images of hand trajectories (here we consider 6 time frames). As evident in the figure, the hypothesis that one is reaching for a small (or big) object entails the hypothesis that the hand will be configured in a precision grip (or power grasp) during action execution – and this hypothesis can be tested during action observation.

The computational model is described in the next three subsections. The first (*architecture*) rehearses active inference and its essential variables, see Fig. 1A. The second (*generative models*) describes the generative models of the two grasping actions (precision grip to a small object or power grasp to a big object) that predict the unfolding of hand movement kinematics and updating the saliency map (Fig. 1B). The third (*hypothesis testing*) describes how the two competing perceptual hypotheses (the actor reaching for a big or a small object, see Fig. 1C) are encoded and tested by saccadic sampling of the most salient elements of the visual scene, and the saliency map that underwrites this epistemic foraging.

### 2.1 Architecture

The architecture of the computational model is sketched in Fig. 1. It follows the hierarchical form for generalized predictive coding (Friston, 2010), where state and error units (black and red units, respectively) are the variables of the systems and are partitioned into cause (*v*), hidden (*x*) and control (*u*) states; and the tilde notation $\tilde{\mu }$ denotes variables in generalized coordinates of motion $\left(\mu ,\mu^{\prime },\mu^{\prime \prime },\ldots \right)$ (Friston, 2008). In the generative model, causal states link hierarchical levels (i.e., the output of one level provides input to the next); hidden states encode dynamics over time; and hidden controls encode representations of actions that affect transitions between hidden states. It is these control states from which actions (e.g., saccades) are sampled.

At the first hierarchical layer of the architecture, sensory signals $\left(v^{\left(0\right)}:=s\right)$ are generated in two modalities: proprioception (*p*) and vision (*q*):

• Proprioceptive signals, encoded in $s_{p}\in {\mathbb{R}}^{2}$, represent the centre of gaze and have an associated (precision-weighted) prediction error $\xi_{v,p}$; i.e., the difference between conditional expectations and predicted values.
• Visual signals, encoded in an array of $s_{q}\in {\mathbb{R}}^{256}$, sample a visual scene uniformly with a grid of 16 × 16 sensors, and have an associated (precision-weighted) prediction error $\xi_{v,q}$.

### 2.2 Hidden states include

• Proprioceptive internal states, which encode an internal representation of the centre of oculomotor fixation. Their corresponding expectation (i.e., neuronal activity) is denoted as ${\tilde{\mu }}_{x,p}\in {\mathbb{R}}^{2}$ and their prediction error as $\xi_{x,p}$.
• Perceptual internal states, encoding the (logarithm of the) probability that each hypothesis is the cause of the visual input. Their corresponding variational mode (i.e., neuronal activity) is denoted as ${\tilde{\mu }}_{x,q}\in {\mathbb{R}}^{N}$ and their prediction error as $\xi_{x,q}$.

Hidden controls $\tilde{u}={\tilde{\eta }}_{u}+{\tilde{\omega }}_{u}$ are modelled as 2D points ${\tilde{\eta }}_{u}$ plus a Gaussian noise perturbation ${\tilde{\omega }}_{u}$, and determine the location that attracts gaze. Their corresponding expectation is denoted as ${\tilde{\mu }}_{u}\in {\mathbb{R}}^{2}$ and their prediction error as $\xi_{u}$.

Action *a* is modelled as classical reflex arc suppressing proprioceptive prediction errors and producing saccadic movements by solving the following equation: $\dot{a}=-\frac{\partial \tilde{s}}{\partial a}\xi_{v}^{\left(1\right)}$. Defining $q\left(\tilde{x},\tilde{v},\tilde{u}|{\tilde{\mu }}_{x}\left(t+\tau \right),{\tilde{\mu }}_{v}\left(t+\tau \right),{\tilde{\eta }}_{j}\right)$ as the conditional density over hidden controls, parameterized by hidden states and causes in the future, salience *S* can be defined as the negentropy (inverse uncertainty) of the conditional density *q*:

$$S\left({\tilde{\eta }}_{j}\right)=-H\left[q\left(\tilde{x},\tilde{v},\tilde{u}|{\tilde{\mu }}_{x}\left(t+\tau \right),{\tilde{\mu }}_{v}\left(t+\tau \right),{\tilde{\eta }}_{j}\right)\right]$$

Thus, the system aims to find the (eye) control that maximizes salience; i.e.,

$${\tilde{\eta }}_{u}=\underset{\tilde{\eta_{j}}}{\text{argmax}}S\left(\tilde{\eta_{j}}\right)$$

Or, more intuitively, sampling the most informative locations (given the current agent's belief state).

### 2.3 Generative models

The computational scheme introduced so far is generic and implements active sampling of information in a variety of perceptual tasks (Friston et al., 2012). In this paper, we use it for an action observation task (Ambrosini et al., 2011), in which the agent (observer) has two hypotheses about the hidden causes of visual input. These hypotheses correspond to reaching for a big object (with a power grip) or reaching for a smaller object in a nearby location (with a precision grip). To test these competing hypotheses, the architecture needs to generate predictions about the current and future sensory outcomes (i.e., observed hand movements and configurations). These predictions are generated from a forward or generative model of reach-to-grasp actions, enabling one to accumulate evidence for different hypotheses – and to evaluate a salience map for the next saccade (see below). In keeping with embodied and motor cognition theories, we consider these generative models to be embodied in the so-called action observation brain network, a network of sensorimotor brain regions that may support action understanding via the simulation of one's own action (Dindo et al., 2011, Friston et al., 2011, Grafton, 2009, Kilner et al., 2007, Pezzulo, 2013) and that includes both cortical and subcortical structures (Bonini, 2016, Caligiore et al., 2013), see also Fig. 1B.

For simplicity, we implemented four generative sub-models predicting the location and configuration of the hand (preshape) under the two hypotheses (reaching for a big or small object) separately. This allows the agent to accumulate sensory evidence in two modalities (hand position and configuration) for each of the two hypotheses. Furthermore, these sub-models provided predictions of hand position and configuration in the future, under the two hypotheses in question.

**Fig. 2:**
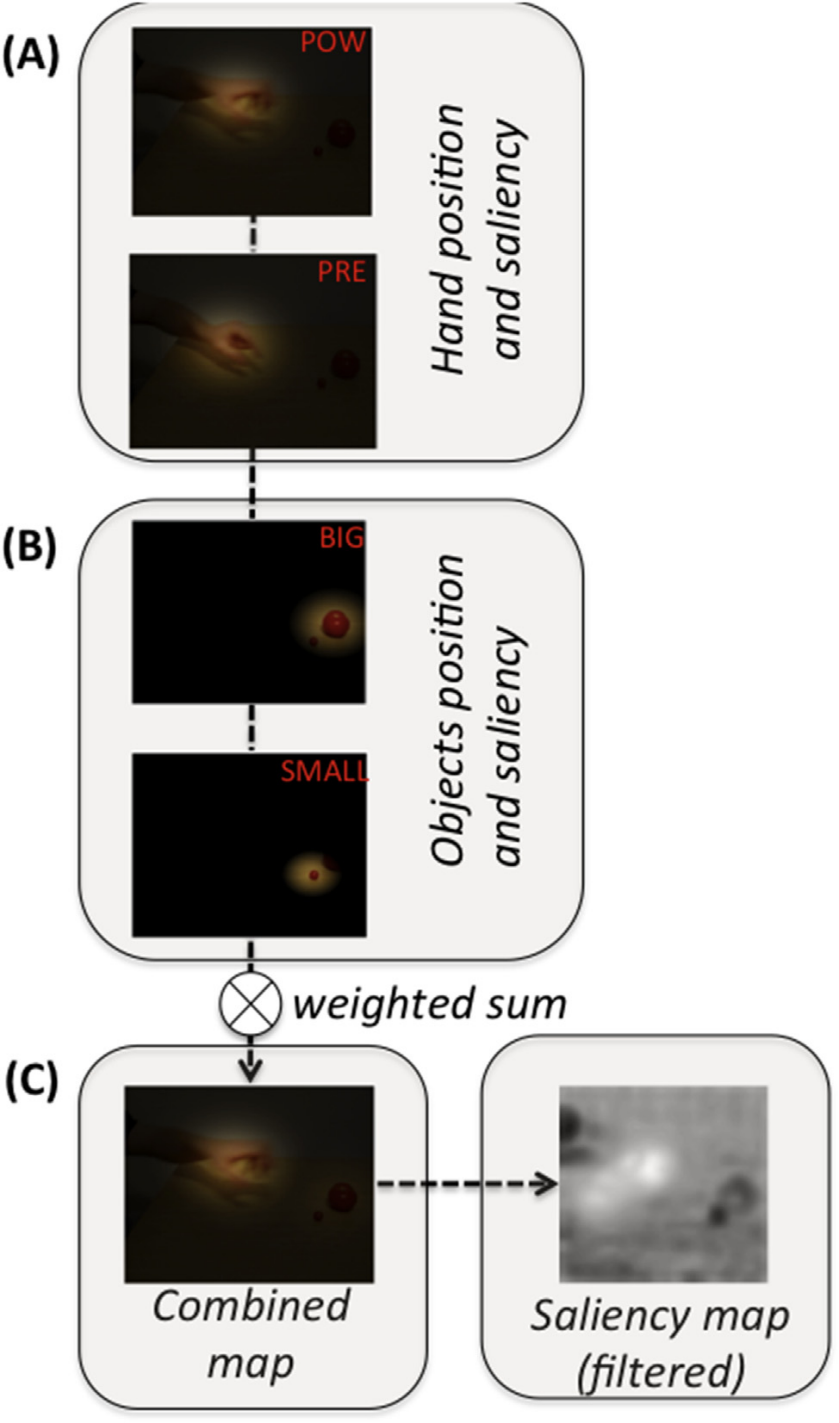
**Graphical representation of how the (pulvinar) saliency map used for the simulations is computed**. The map is the linear combination of four maps. (A) Each of the first two maps represents the (expected) hand position under the two hypotheses (POW is power grasp, PRE is precision grip), and the corresponding saliency. In the POW (or PRE) map, hand position is represented as a Gaussian, whose centre is computed by a *forward model of hand position*, conditioned on the power grasp (or precision grip) hypothesis. The weight assigned to the POW (or PRE) map in the computation of the saliency map (see below) is the probability of power grasp (or precision grip) as computed by a *forward model of preshape information*, conditioned on the power grasp (or precision grip) hypothesis. (B) The second two maps represent the position and saliency of the two objects (BIG or SMALL), given the current belief state of the agent. The position of the BIG (or SMALL) object is different but fixed for each trial. It is represented as a Gaussian centred on the object position. The weight assigned to the BIG (or SMALL) map in the computation of the final saliency map (see below) depends on both the posterior probability that the BIG (or SMALL) object multiplied by a term that reflects the current distance between hand and BIG (or SMALL) position. (C) The resulting saliency map is obtained as the weighted combination of the four aforementioned maps. This map is filtered to be used by the system. (Note that the saliency map shown here is an illustrative example, not a superimposition of the four components).

These four probabilistic sub-models were learned on the basis of hand movement data collected from six adult male participants. Each participant executed 50 precision grip movements directed to a small object (the small ball) and 50 power grasp movements directed to a big object (the big ball), and data on finger and wrist angles were collected using a dataglove (HumanGlove – Humanware S.r.l., Pontedera, Pisa, Italy) endowed with 16 sensors (3 angles for each finger and 1 angle for the wrist). The four sub-models used in the simulations were obtained by regressing the aforementioned data (300 trials for each sub-model), to obtain probability distributions over the angles of the fingers and wrist, over time. To regress each sub-model, we used a separate Echo State Gaussian Processes (ESGP) (Chatzis & Demiris, 2011): an algorithm that produced a predictive distribution over trajectories of angles, under a particular sub-model, see Fig. 2A. The ESGP sub-models were trained off-line to predict the content of the next frame of the videos used in the experiments (6 frames) and to map the angles of the fingers and wrist to the visual appearance (preshape) and position in space of the hand, respectively.

After the off-line learning phase, the four forward sub-models generate a probabilistic prediction of the next hand preshape and position based on all previous sensory images. This enables the probability of the two competing hypotheses to be evaluated, using the method described in Dindo et al. (2011).

More formally, the first two sub-models encode the trajectories traced by subjects' hands during the trials, thus predicting the probability of the hand position in the image (as Gaussians) under the hypothesis of grasping a small object (SMALL):

$$p^{SMALL}\left(hPos\left(t\right)\right)=p\left(hPos\left(t\right)|hPos\left(t-1\right),G=SMALL\right)$$

and grasping a big object (BIG):

$$p^{BIG}\left(hPos\left(t\right)\right)=p\left(hPos\left(t\right)|hPos\left(t-1\right),G=BIG\right)$$

respectively.

Analogously, the second two sub-models encode the probability of the hand configuration (preshape) in the image under the hypothesis of grasping a small object (SMALL):

$$p^{SMALL}\left(hShape\left(t\right)\right)=p\left(hShape\left(t\right)|hShape\left(t-1\right),G=SMALL\right)$$

and grasping a big object (BIG):

$$p^{BIG}\left(hShape\left(t\right)\right)=p\left(hShape\left(t\right)|hShape\left(t-1\right),G=BIG\right)$$

respectively.

Similarly, we encode the positions of the two objects, small:

$$p^{SMALL}\left(gPos\left(t\right)\right)=p\left(gPos\left(t\right)|gPos\left(t-1\right),G=SMALL\right)$$

and big:

$$p^{BIG}\left(gPos\left(t\right)\right)=p\left(gPos\left(t\right)|gPos\left(t-1\right),G=BIG\right)$$

respectively. Note that for generality (and notational uniformity) these are written as if they were a function of time. However, objects have fixed positions during a trial; hence it is not necessary to use an ESGP to calculate them.

In summary, we used a sophisticated (Echo-state Gaussian process) model to generate predictions in two modalities and thereby accumulate evidence for the two competing hypotheses. The inversion of this forward model (or models) is formally equivalent to Bayesian filtering or predictive coding, but using a more flexible and bespoke generative model. In turn, we will see below that the posterior beliefs (about location and configuration of the hand and location of the target object) are used to form Bayesian model averages of the salience maps under competing hypotheses.

### 2.4 Hypothesis testing

Our action observation task can be described as a competition between two alternative hypotheses (power grasp to the big object *vs* precision grip to the small object). Importantly, saccades are treated as “experiments” that gather evidence in favour of each hypothesis – so that they can be disambiguated. Given that this is a dynamic task and actions unfold in time, the two competing hypotheses have to explain *sequences* of images, and not a single frame; in other words, they have to explain the whole trajectory and not just the final hand position: see Fig. 1C. This calls for sequential hypothesis testing as the observed action unfolds.

The target of the next saccade is sampled from a saliency map (see Fig. 1A), which evaluates the (epistemic and pragmatic) value of sampling each location in the visual scene – and is continuously updated during action observation. The salience map comprises the Bayesian model average of four component salience maps, based on local samples of the visual field (modelled with Gaussian windows): see Fig. 2. For the hand salience map (Fig. 2A), we used the Bayesian model average under the four sub-models generating position and configuration, under reaching for big and small objects, respectively. This captures the fact that the value of locations where the agent expects to find a hand configured for a power grasp or precision grip increases in relation to the estimated probability of reaching the big or the small object. For the object salience map (Fig. 2B), we used a Bayesian model average of Gaussian windows centred on the object (which is fixed), weighted by the probability of reaching big or small object and the relative hand-object distance. This captures the fact that the identity of the target object resolves more uncertainty about the intended movement when the hand is closer; i.e., approaching the object. Finally, the hand and object salience maps were combined and downsampled (using on-off centre-surround sampling) to obtain a smaller (16 × 16 grid) saliency map that is computationally more tractable (Fig. 2C). Note that for clarity the combined map shown in Fig. 2C is illustrative and it is not the true superimposition of the four images above.

In detail, we compute $S_{k}=S\left({\tilde{\eta }}_{u}\right)-\text{min}\left(S\left({\tilde{\eta }}_{u}\right)\right)$, the differential salience for the *k*th saccade and enhance it by $R_{k}$, i.e., ${\bar{S}}_{k}=S_{k}+R_{k}$ with $R_{k}$ corresponding to the map

$$R_{k}=\sum_{j=1}^{4}w_{j}\rho \left(S_{k};c_{j}\right)+a\cdot R_{k-1}$$

with *a* representing the weight of previous estimates, which is set to 1/2 for coherence with (Friston et al., 2012). The elements of the equation are computed on the basis of the preceding ESGP models:

• ρ is a Gaussian function (with a standard deviation of 1/16 of the image size) of the distance from the points $c_{j}$;
• $c_{1}∼p^{SMALL}\left(hPos\left(t+1\right)\right)$ and $c_{2}∼p^{BIG}\left(hPos\left(t+1\right)\right)$ are predicted points of the position of the hand;
• $c_{3}∼p^{SMALL}\left(gPos\left(t+1\right)\right)$, $c_{4}∼p^{BIG}\left(gPos\left(t+1\right)\right)$ are predicted points of the goal position;
• $w_{1}=p\left(G=SMALL|hShape\left(1:t\right)\right)$ and $w_{2}=p\left(G=BIG|hShape\left(1:t\right)\right)$ are predictions of grasping action computed on the basis of the hand preshape models;
• $w_{3}=p\left(G=SMALL|OBS\left(1:t\right)\right)$ and $w_{4}=p\left(G=BIG|OBS\left(1:t\right)\right)\;$ are beliefs about the currently performed grasping action.

where OBS(1:t) denotes the sequence of previous observations.

The coefficients of the map and the relative salience of the elements within it (hand and objects) depend on the outputs of the generative models described earlier. For the hand salience maps, the centre of Gaussians was based on the forward models of hand position under the precision grip (or power grasp) hypothesis, while the “weight” of the map $w_{1}$ (or $w_{2}$) is calculated based on the forward model of preshape information under the precision grip (or power grasp) hypothesis. In other words, salience of hand position expected under the precision grip (or power grasp) hypothesis is higher when the hand is correctly configured for a precision grip (or power grasp). This is because, in the empirical study we are modelling, only preshape depends on the performer's goal (while hand position is uninformative); however, the same model can be readily expanded to integrate (in a Bayesian manner) other sources of evidence; such as the actor's hand position and gaze (Ambrosini, Pezzulo, & Costantini, 2015). Furthermore, the salience of the small (or the big) object, and the “weight” of the map $w_{3}$ (or $w_{4}$), corresponds to the probability that the performer agent is executing a precision grip (or power grasp), given the current observations. Specifically, it is calculated as the posterior probability of the small (or big) object hypothesis multiplied by a Gaussian term *N(hPos; gPos,σ)* that essentially describes hand-object distance. The Gaussian is centred on the object position (*gPos*) and *hPos* is the hand position. The *σ* of the Gaussian is the uncertainty about the posterior probability of the small (or big) object hypothesis. Overall, $R_{k}$ represents a dynamic (and fading) snapshot of the current belief of the perceived action based on the observation of the trajectories and preshape of the subjects' hands.

The most salient zones of the saliency map of Fig. 2C represent the most informative locations of the visual scene; i.e., those that are expected to disambiguate alternative hypotheses. Therefore, the map does not simply include spatial information (e.g., the expected position of the hand), but also information about the (epistemic) value of the observations (e.g., a hand preshaped for power grasp) one can harvest by looking at these positions, given the current belief state of the agent. Hence, hypothesis testing – or the active sampling for the most relevant information – corresponds to selecting the most salient location for the next saccade. Note that this is a dynamical process: the saliency map is continuously updated reflecting the changing beliefs of the agent.

### 2.5 Modelling perceptual decisions in action observation

In the action observation paradigm we simulated, participants were not explicitly asked to decide (between “small” or “big” hypotheses) but their “decision” was inferred by measuring their gaze behaviour; i.e., saccade towards one of the two objects, big or small (Ambrosini et al., 2011). In the same way, in the computational model, updates of the agent's belief and saliency map terminate when the (artificial) eye lands on one of the two objects – signalling the agent's decision. As we will see, in both the human experiment and the model, with sufficient information, saccades can be proactive rather than just tracking the moving hand, and participants fixate the selected target before the action is completed.

Note that, in the model, the decision (i.e., the fixation to the selected object) emerges naturally from saliency dynamics, which in turn reflect belief updating during hypothesis testing, without an explicit decision criterion (e.g., look at the big object when you are certain about it). This is because actions are always sampled from the same salience map, which implicitly indicates whether the hand or one of the objects is most contextually salient. In other words, the decision is made when the target location becomes more salient than the other locations (e.g., the hand location), not when the agent has reached a predefined criterion, e.g., a fixed confidence level. This lack of a “threshold” or criterion for the decision marks an important difference with common place models of decision-making such as the drift diffusion model (Ratcliff, 1978) and is a hallmark of embodied models of choice that consider action and perception as mutually interactive rather than modularized systems (Lepora & Pezzulo, 2015).

Key to this result – and the implicit shift from hand-tracking to the fixation of the selected object – is the fact that the posterior probability that one of the two objects will be grasped is continuously updated when new visual samples are collected and can eventually become high enough to drive a saccade (i.e., one of the objects can assume more salience than the hand). This, in turn, depends on the fact that when the probability of a power versus precision grip is updated (Fig. 2A) the probability of the big versus small object is also updated (Fig. 2B), reflecting the implicit knowledge of the intentionality of the action (e.g., that big objects require a power grasp). In sum, if the agent does not know the goal, as in this perceptual paradigm, it has to accumulate evidence first by looking at the hand, and then by looking at the target when it has resolved its uncertainty.

**Fig. 3:**
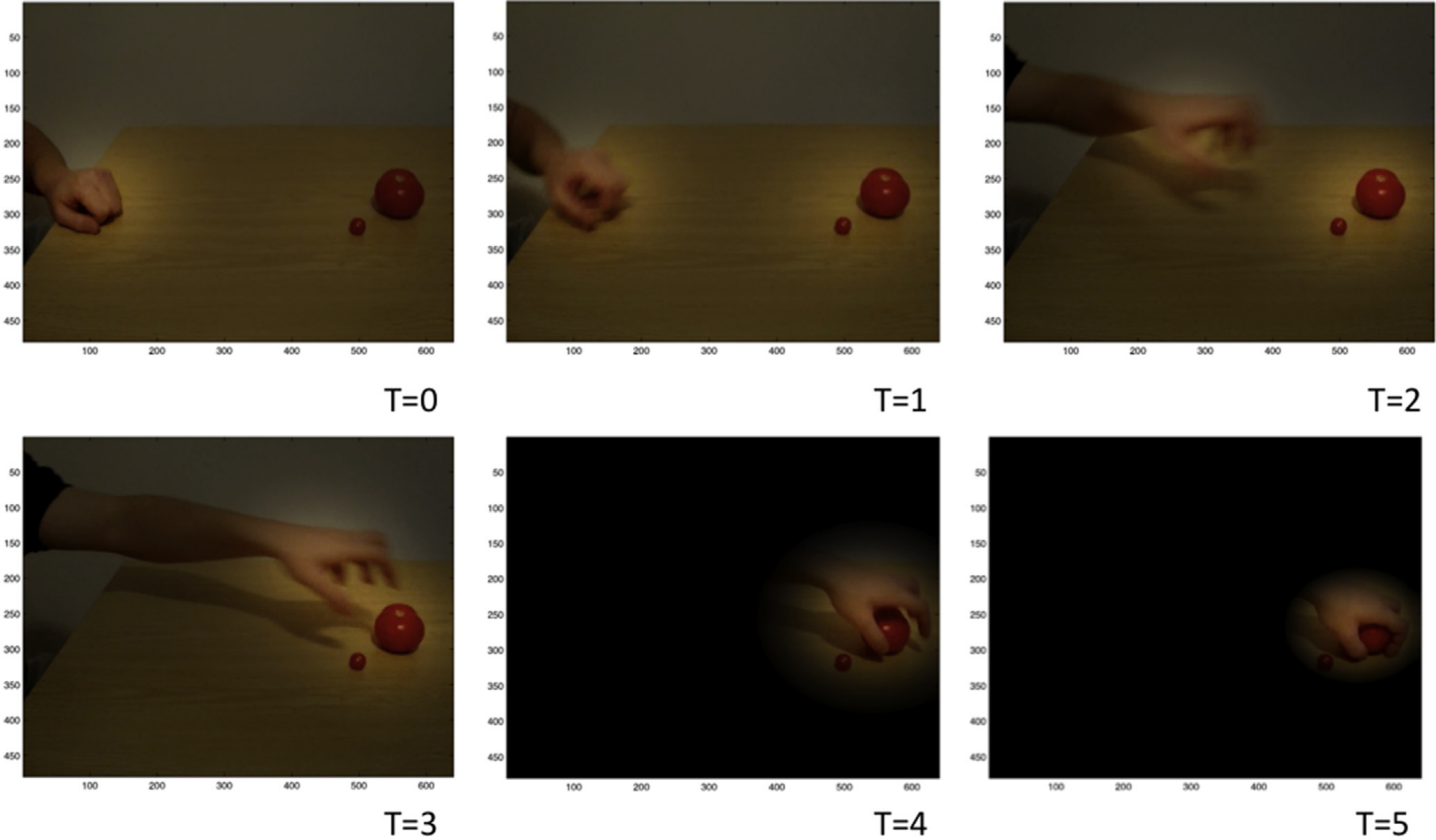
**A sample saliency map, shown during 6 time frames**. The figure shows how the saliency map (as in Fig. 2C) evolves over time as the actors action unfolds. This map encodes perceptual aspects of the observed scene (e.g., hand position and configuration) as well as the expected informational or epistemic value (salience) of the percept. Bright areas correspond to high-saliency locations. Note that the saliency map is updated during action observation, reflecting the changing belief state of the observer or agent. At the time frame T2, the most salient location is the big object. Since actions (gazes) are sampled from the most salient locations in the saliency map, the agent is more likely in it a proactive saccade to the big object, even if the hand has not yet reached it.

As an illustrative example, Fig. 3 shows a sequence of (unfiltered) saliency maps along the six time frames of a sample run. Here, the brighter areas correspond to the most salient locations (recall that the most salient area is selected for the next saccade). One can see a shift in the saliency map, such that, by the third frame, the most salient object is the to-be-grasped big object. Below we test the behaviour of the model by directly comparing it with human data.

## 3 Results

We tested the computational model on the visual stimuli used by Ambrosini et al. (2011), which include action observation in four (2 × 2) conditions, which derive from the combination of 2 target conditions (big or small object) and 2 shape conditions (pre-shape or no-shape). As a result, the four conditions correspond to four types of hand actions: “no-shape–big target”, “no-shape–small target” (i.e., a hand movement with the fist closed to the big or small target, respectively), “pre-shape–big target”, and “pre-shape–small target” (i.e., a hand movement with a power grasp or a precision grip to grasp the big or small object, respectively).

To compare the results of the original study and the simulations, we calculated the arrival time for the simulated saccades as the difference between the time when the hand (of the actor) and the saccade (of the simulated agent) land on the target object. Note that arrival time is negative when the eye lands on the object before the hand. Note also that our simulations include one simplification: saccades have a fixed duration (of 192 msec, which stems from the fact that before a saccade the inference algorithm performs 16 iterations, each assumed to last 12 msec). These parameters were selected for consistency with previous work using the saccadic eye movement model (Friston et al., 2012) and to ensure that the simulated saccadic duration is within the average range for humans (Leigh & Zee, 2015). Given that both saccades and videos have fixed duration, every trial comprises exactly 6 epochs.

**Fig. 4:**
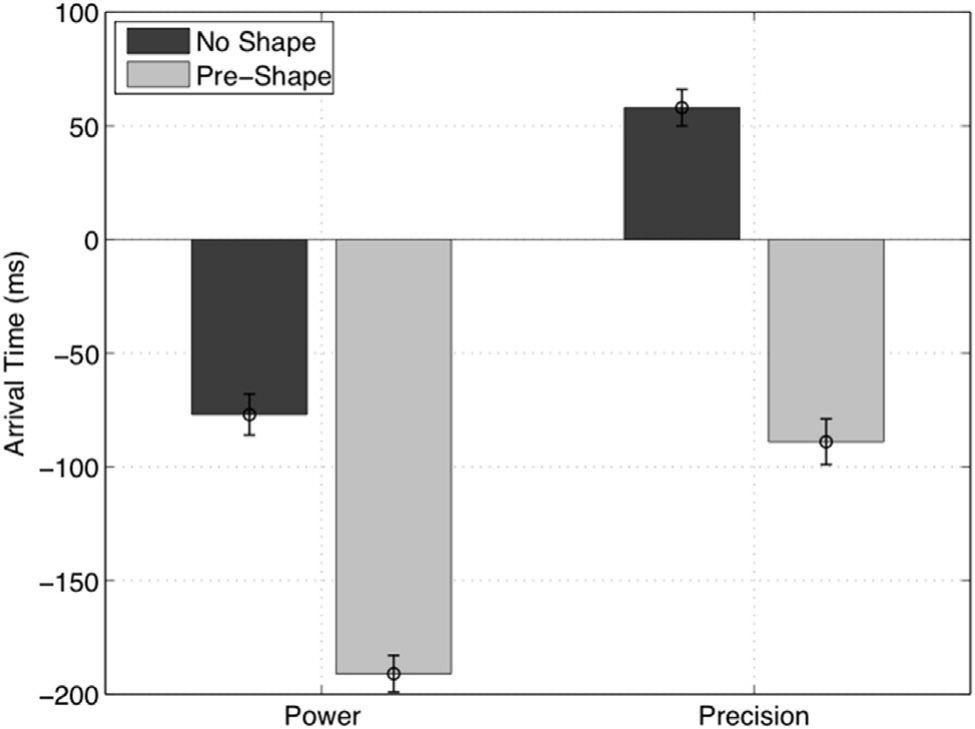
**Results of the simulations, arrival time**. Every iteration lasts 12 msec. For simplicity, saccades are assumed to have a fixed duration of 16 × 12 = 192 msec. Arrival time is calculated as the difference between the time when the hand (of the actor) and the eye (of the participant) land on the object, as in the original study of Ambrosini et al. (2011). It is negative when the eye lands on the object before the hand. Note that arrival times for the big object (power grasp) are more anticipatory than for the small object (precision grip). This phenomena was also observed in the simulations (compare Fig. 5, Fig. 6).

The results of our simulations are remarkably similar to those of the original study (Fig. 4). The key result is a significant advantage for the pre-shape over the no-shape condition, for both power grasp and precision grip. This result stems from the fact that in the pre-shape, information about the actor's goal can be inferred from the hand movement kinematics, enabling an anticipatory saccade to the target to confirm the agent's (or participant's) beliefs.

**Fig. 5:**
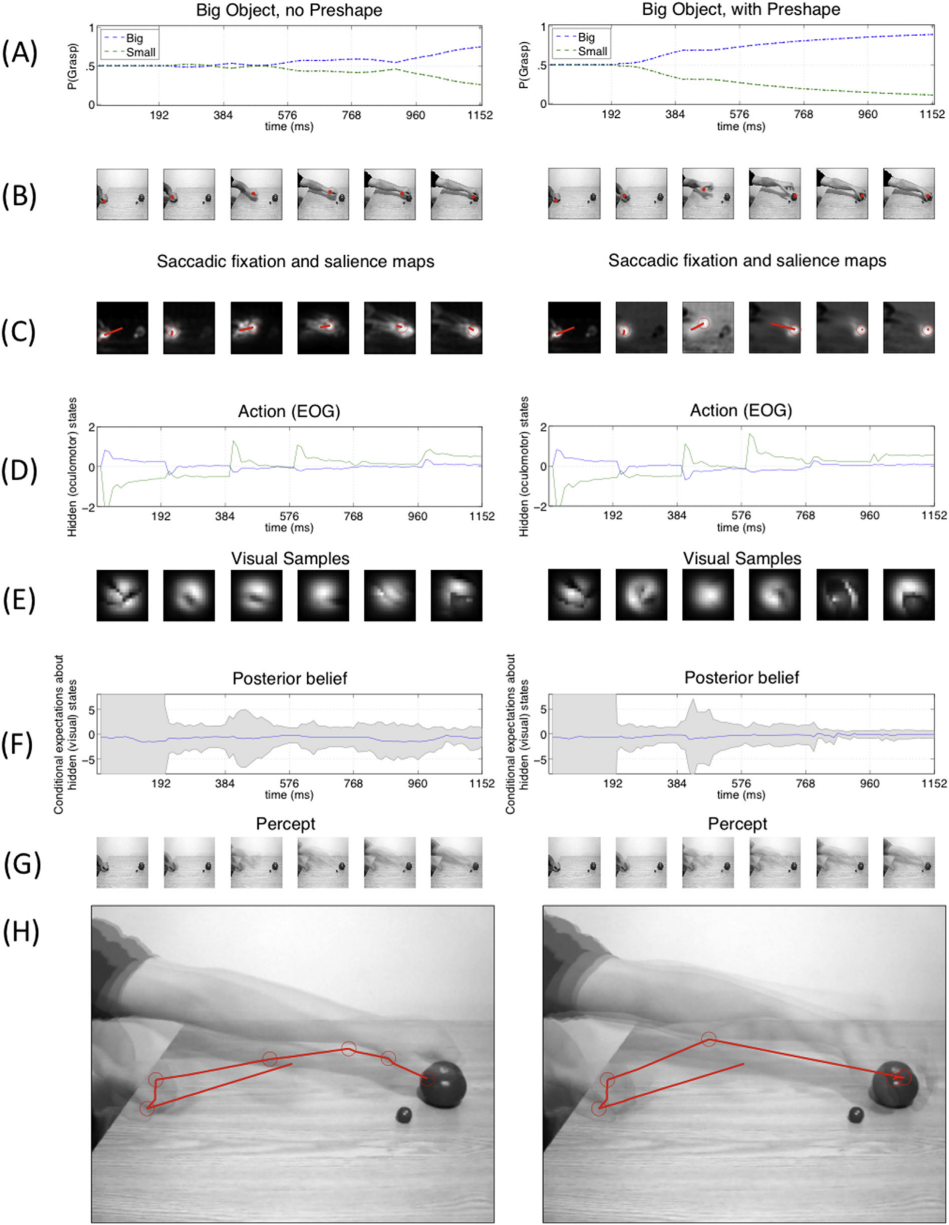
**Results of sample simulations of power grasp, without preshape (left) or with preshape (right)**. Panel A shows the expected probability of the two competing hypotheses (here, big *vs* small) during an exemplar trial. Panels B and C show the location of the saccade in the video frame and the saliency map, respectively. Panel D shows the hidden (oculomotor) states as computed by the model. Panel E show the actual content of what is sampled by a saccade (in the filtered map). Panel F shows the posterior beliefs about the ‘true’ hypothesis (expectations are in blue and associated uncertainty are in grey). The posterior beliefs are plotted in terms of expected log probabilities and associated 90% confidence interval. A value of zero corresponds to an expected probability of one. Increases in conditional confidence about the expected log probability correspond to a shrinking of the confidence intervals. Panel G show the “percept” of the system – that is, the mixture of hypotheses weighted by the posterior expectation, which in this study is represented with a superposition of all the frames of the previous time steps. Panel H shows the sequence of saccades during the experiment (where the first saccade to the hand depends on participants' instructions and can be ignored, see the main text). Note that in the (left) case without preshape, gaze follows a reactive, hand-following strategy (panels G and H) and the action is disambiguated fairly late in the trial (panels A and F). The scenario is different in the (right) case with preshape information.

**Fig. 6:**
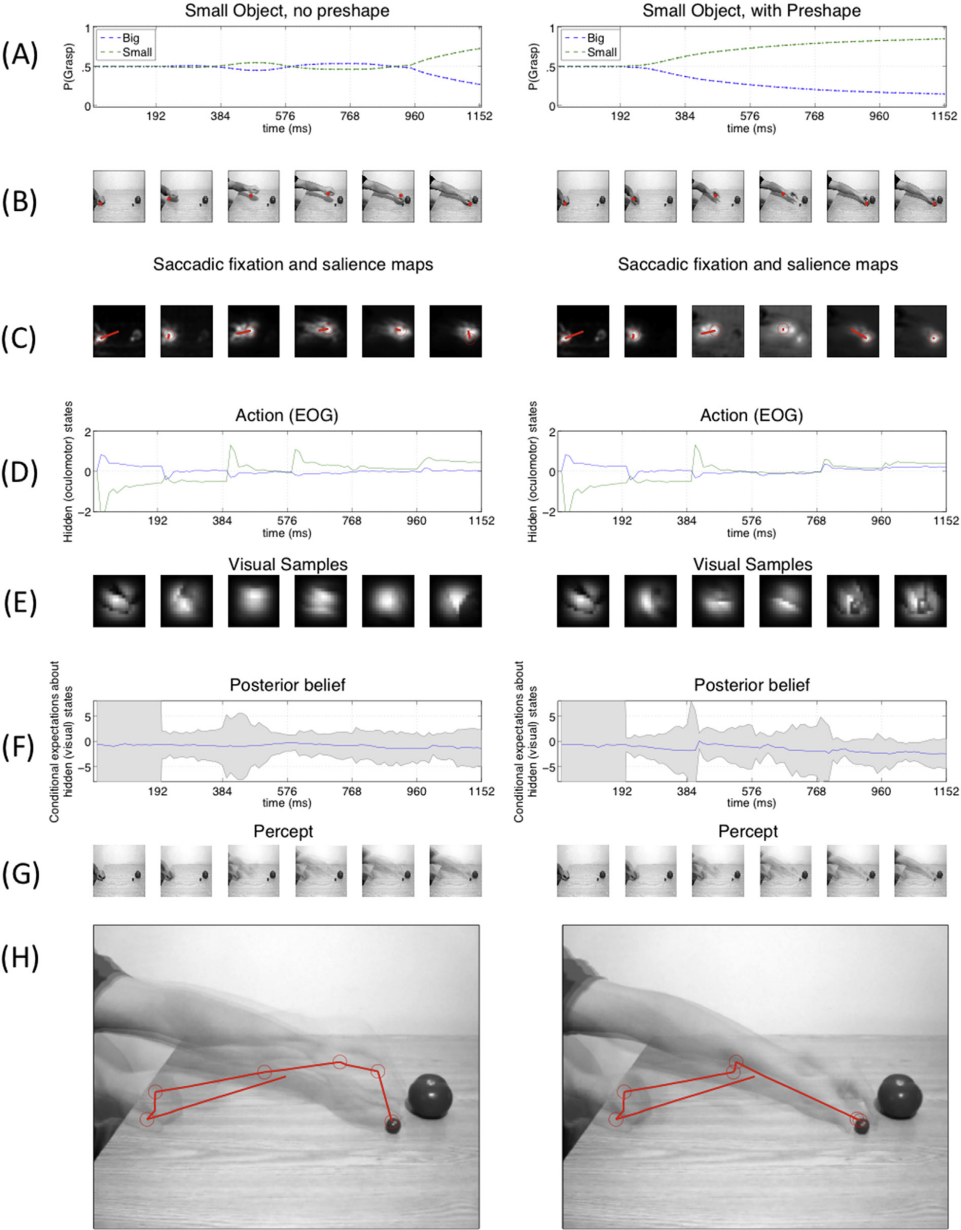
**Results of sample simulations of precision grip, without preshape (left) or with preshape (right)**. Labels as in Fig. 5. Note that even in these sample simulations, the (right) scenario with preshape entails faster recognition and proactive movements compared to the (left) scenario without preshape.

This difference can be appreciated by looking at Fig. 5, Fig. 6, which show sample simulations for each of the four experimental conditions. Fig. 5 shows side-by-side exemplar simulations of power grasp without preshape (left) and with preshape information (right). Fig. 6 shows side-by-side example simulations of precision grip without preshape (left) and with preshape information (right). Panels A of Fig. 5, Fig. 6 report the probability of the two competing hypotheses (here, big *vs* small, aka power grasp *vs* precision grip) during observation. One can see that in the condition without preshape, the probability of the two hypotheses only becomes significantly different late in the trajectory.

Furthermore, we observe a significant difference between a reactive hand-following gaze strategy, which emerges in the no-shape condition, and an anticipatory gaze strategy, which emerges in the pre-shape condition, shortly after the beginning of a trial. This difference is evident if one considers panels B and C of Fig. 5, Fig. 6, which show the location of the saccade in the video frame and the saliency map, respectively; and panels I of the same figures, which show the sequence of saccades during the experiment (note that the first saccade is always from the centre to the initial hand movement. This reflects the fact that in the human experiment, participants were asked to fixate on the actor's hand before watching the video; however, this first saccade was ignored in the analysis). Heuristically, at the beginning of a trial, there is little information in the position of the hand that can inform beliefs about the target. Therefore, the most salient locations to sample are the hand itself, in the hope that its configuration will portend the ultimate movement. However, as time progresses and the hand approaches its target, the identity of the nearest object resolves more uncertainty about the intended movement. One would therefore anticipate saccades to the object at later points in the trajectory, with an implicit reporting of the final belief (or decision) by a saccade to the target object. Clearly, the above strategy will only work when a hand is pre-configured in an informative way. If the configuration of the hand does not emerge (or emerges later) in the trajectory, the hand should be tracked more closely – in search (or anticipation) of an informative change in configuration.

This latter observation highlights the importance of generative models in driving eye movements during action observation. If observed movements do not resolve uncertainty about the performer's action goals, eye movements cannot be proactive. The importance of generative models for proactive eye movements was highlighted in a study by Costantini et al. (2014). The authors used repetitive transcranial magnetic stimulation (rTMS) to induce “virtual lesions” in participants that performed a task equivalent to the one described here. The results of the experiment show that eye movements become reactive when the virtual lesion is applied to the left ventral premotor cortex (PMv) – an area thought to be part of a forward model for action execution. The same study showed that virtual lesions to the posterior part of the STS do not produce equivalent impairments. In predictive coding models of action observation and the mirror neuron system (MNS), STS is considered to lie at a low level of the (putative) MNS hierarchy, possibly coding (highly processed) perceptual aspects of biological motion. This result is thus compatible with the notion that it is specifically the motor-prediction aspect of the generative model that is crucial for hypothesis testing, not (high-order) visual processing; but this interpretation demands more scrutiny in future research.

Finally, in both the original study and our simulations, the “big” hypothesis is discriminated faster than the “small” hypothesis. This may be due to a greater salience of movement kinematics elicited in the context of the power grasp: the ESGP model for power grasp has overall lower uncertainty than the ESGP model for precision grip (compare Fig. 5, Fig. 6F). In other words, both human participants and our models may be sensitive to subtle (and early) kinematic cues that emerge earlier under power grasps. In the original report, it was suggested that this advantage may also have a perceptual nature, and participants may select the big object as their default option (perhaps because it is more perceptually salient). We tested this notion using a (small) prior probability for the big hypothesis (implemented via a Gaussian centred on .57 with variance .01). This did not influence our results; either in terms of discriminating the big target movements earlier or in terms of the differences in action recognition with and without preshape information.

## 4 Discussion

We have shown that the dynamics of eye movements described by active inference – an extension of the predictive coding formalism to the domain of action – correctly reproduce human behaviour in a series of action observation tasks. This model rests on two intertwined notions. First, action observation can be described formally as *hypothesis testing*, or the active sampling of salient information, as informed by the agent's predictions. Second, the process relies on an *internal generative model* that generates predictions of the next sensory sample (conditioned on the agent's competing perceptual hypotheses). These two components act synergistically: the predictions of the generative model are used to update a visual *saliency map*, which serves to direct saccades to the most informative parts of the visual scene (active sampling). In turn, active sampling provides evidential input to the generative model, which is used to update the predictions and the probability of competing hypotheses.

The first innovative aspect of our proposal entails casting action observation as the inferential process of *hypothesis testing* and not (for example) as a classification or resonance mechanism, or one in which the observing agent passively receives (rather than actively sampling) information. Our hypothesis thus contrasts with models that describe action understanding as a resonance (Rizzolatti et al., 1996) or a Hebbian (Keysers & Perrett, 2004) mechanism. It also contrasts with accounts of action observation as a purely visual recognition task (Fleischer et al., 2013). Our proposal is related to various models that include predictive mechanisms and forward models for action understanding (Demiris, 2007, Dindo et al., 2011, Donnarumma et al, 2017, Wolpert et al., 2003) and concomitant attentional allocation (Demiris and Khadhouri, 2005, Ognibene and Demiris, 2013). However, our model significantly differs from all the aforementioned models, because, first, it uses eye movements and hypothesis testing in the action understanding process, and second, it adopts an active inference scheme that dispenses with any form of inverse model (Friston, 2011). From a broader perspective, one can consider the perceiver's actions to be essential for action understanding and, more generally, to cognitive processing at large (Engel et al., 2013, Pezzulo, 2008, Pezzulo, 2011, Pezzulo and Cisek, 2016). Here, two kinds of actions are essential for action understanding: overt and covert. The former are the eye movements and the saccades that are used as “experiments” that permit hypothesis testing; that is, active perception (Bajcsy et al., 2016, Gibson, 1966). The latter are the covert reactivations of the sensorimotor system (and its generative model) that permit generating predictions; that is, motor cognition (Jeannerod, 2006, Pezzulo et al., 2011).

This latter point leads us to a second important aspect of our proposal. Our account of action observation is in keeping with embodied and motor cognition theories, especially if the generative model used for hypothesis testing is used for performing goal-directed actions (Flanagan & Johansson, 2003). In the social domain, support for this view comes from a variety of sources, including studies of motor activation during action observation, or interference effects between observed and performed actions (Aglioti et al., 2008, Cross et al., 2006, Kilner et al., 2003, Umiltà et al., 2001), see Kilner and Lemon (2013) for a review. This sort of evidence has motivated a variety of theoretical and computational models of motor involvement in action observation (Demiris and Khadhouri, 2005, Friston et al., 2011, Ognibene and Demiris, 2013, Ognibene et al., 2013, Wolpert et al., 2003), see Giese and Rizzolatti (2015) for a review. Our model significantly advances the state of the art by assigning the motor system a role in hypothesis testing during action observation, too. Direct support for this idea, which is rarely addressed in models of action observation, comes from studies that show that saccades essentially cease to be predictive and most often simply follow the moving hand in various conditions that prevent the recruitment of the motor system: when the motor system is compromised with TMS (Costantini et al., 2014) or engaged in an interfering task (Costantini, Ambrosini, & Sinigaglia, 2012), when hands are tied (Ambrosini, Sinigaglia, & Costantini, 2012), or the perceiver does not include the observed action in her repertoire (Ambrosini et al., 2013).

A third innovative aspect of our proposal is that it parsimoniously explains eye movements dynamics as the emergent effect of using (and updating) a saliency map – that is, a domain-general mechanism that dispenses from any ad-hoc or task-specific criterion (e.g., a decision threshold or criterion). In this respect, it is important to emphasize that the notion of a saliency map does not reduce to a series of filters (or other mechanisms) that capture perceptual features in a bottom-up way, as usually assumed in the literature. Rather, the contribution of top-down, hypothesis-driven predictions is essential in updating the content of the map (e.g., which objects are expected and where) and the saliency assigned to each location (e.g., how important is a saccade to each location for testing the current hypotheses) – which is in keeping with theories that highlight top-down processes in visual perception (Hayhoe and Ballard, 2005, Rothkopf et al., 2007, Tatler et al., 2013; see also Corbetta & Shulman, 2002). The possibility to assign salience to locations that encode goals (for action execution or observation) distinguishes this approach from alternative proposals that focus on the information gain afforded by low-level properties of the visual stimuli (Itti & Koch, 2000).

Furthermore, our approach entails a *systems-level* perspective on action understanding. The importance of brain mechanisms such as mirror neurons in action recognition has been often recognized, but clearly these neurons (like any other neuron) operate within much larger brain networks for adaptive action and perception. This implies the necessity of a systems-level view of action recognition, which clearly recognizes the role of large cortical areas and cortico-subcortical loops (Bonini, 2016, Caligiore et al., 2013). The systems-level architectural scheme of Fig. 1 – despite it is necessarily simplified and incomplete – represents a first step in this direction. Addressing action understanding within a large-scale biological model like active inference permits to generate specific predictions on the role of different brain areas in this process.

Finally, it is worth highlighting that we have tested the validity of the model at the behavioural level, and its capacity to explain different patterns of (proactive or reactive) eye movements by appealing to a single imperative of uncertainty (i.e., expected surprise or variational free energy) minimization. Clearly, there are several other aspects of the proposal that remain to be tested in more detail. One advantage of our computational approach is that it enables the estimation of *hidden variables* from behavioural data. For example, panels D and F of Fig. 5, Fig. 6 show the hidden (oculomotor) states and the agent's current uncertainty, respectively. These measures (and others) are automatically inferred by the model and can be used for model-based, trial-by-trial computational analysis of neurobiological data, such as for example dynamical measures of brain activation such as EEG or MEG (Daw, 2011, Friston et al., 2014), thus productively linking various levels and timescales of action observation, behavioural and neuronal. This reflects the fact that the proposed model generates a variety of empirical predictions, concerning for example the ways action – or belief-related brain signals (Panels D and F in Fig. 5, Fig. 6) – change during trials with high or low uncertainty, or when the motor system is temporarily inactivated (Costantini et al., 2014), which can be tested empirically. Another prediction is that, because action understanding is an active process, modulations of the hypothesis testing mechanism would influence it; for example, that it would be possible to bias action understanding by restricting eye movements.

Compared to the original model of Friston et al. (2012), there are three main differences. The first difference is the fact that the perceptual stimulus is dynamical (a video and not an image), and for this, the two perceptual hypotheses correspond to image sequences not images. The second difference lies in the way the saliency map is computed – here, it does not depend on perceptual features of the to-be-recognized objects but on motor predictions. The third important difference between the current scheme and that described by Friston and colleagues is that we eschew an ad hoc inhibition of return – which they included because their generative model did not have any memory. This meant that the simulated agent forgot what it had learned from sampling a previous location and would keep on returning to the most salient visual features in the absence of inhibition of return. Our more realistic setup precludes this because the model generates trajectories that unfold over time. This means that what was salient on the previous saccade is usually less salient on the subsequent saccade. This follows from the fact that our generative model encodes trajectories and therefore has an implicit memory, in the sense that it can accumulate information over time about the underlying causes of sensory information.

The idea of a reuse of motor strategies to support perceptual functions has been raised in several domains. One early example was (the motor theory of) speech perception (Liberman and Mattingly, 1985, Liberman et al., 1967). Our proposal here is in accordance to one central claim of this and other motor theories of cognition (Jeannerod, 2006), namely, that perceptual processing reuses the generative or forward models implied in motor control. In our study, however, the contribution of generative models (and the motor system) is quite specific: guiding eye movements and supporting active hypothesis testing. As our simulations and the experimental data show, engaging the generative models is not mandatory for action recognition, but improves it by making eye movements more proactive. In other words, our simulations show that one can assign saliency to current stimuli (observed movements) and solve the same task in various ways: reactively (by following the hand), by extrapolating perceptual variables over time, or by engaging the generative models (and the motor system). However, reactive strategies may be limited and visual extrapolation may fail to correctly represent sequential events that are generated by hidden causes (e.g., the dynamics of the motor system) and have an intrinsic intentionality; otherwise, the generative model underlying visual extrapolations would be essentially a duplicate of the generative model underlying action execution. Another problem with a visual extrapolation explanation is that it is not immediately clear why eye movements should go proactively to the object (and not, for example, any future predicted location before the object) without a notion that grasping the object is the agent's goal. While it may not be mandatory to engage the (generative model of the) motor system to solve this specific task, doing so would automatically produce an advance understanding of the situation that speaks to one's own action goals (“motor understanding”); in turn, this may have additional benefits such as segmenting action observation in meaningful elements (e.g., goal and subgoal-related ways, Donnarumma et al., 2016, Stoianov et al., 2015) and permitting fast planning of complementary or adversarial actions in social settings (Pezzulo, 2013, Pezzulo et al., 2017).

In this illustration of epistemic foraging under active inference, we have focused on information gain in the context of action observation. On this view, salience becomes a sort of “epistemic affordance”, where the affordance of different locations (hand or objects) changes dynamically as a function of the agent's beliefs – and therefore becomes inherently context sensitive. It is interesting to note that other studies using active inference (but in simplified, Markovian or discrete time scheme) appeal to exactly the same idea, but in the domain of goal-directed action, e.g., finding reward in a maze. In these studies, when agents are uncertain about reward locations, they first need to resolve their uncertainty through epistemic action that entails information gain (e.g., they search for cues that disambiguate a reward location). Resolving this uncertainty is a prerequisite to successively execute a pragmatic action (e.g., reaching the reward location). The resulting mixture of epistemic and pragmatic value turns out to be the free energy expected under any sequence of actions or policy. In short, the active inference we have demonstrated in this work has a construct validity in terms of recent work on more abstract formulations of exploration and exploitation (Friston et al., 2015, Friston et al., 2016a, Friston et al., 2016b, Pezzulo and Rigoli, 2011, Pezzulo et al., 2015, Pezzulo et al., 2016).

## 5 Conclusions

This paper offers a potentially important and novel formulation of action observation that generalizes active inference based on epistemic foraging (foraging for information) and visual salience. In short, we consider the driving force behind saccadic eye movements to be the resolution of uncertainty about competing explanations for the causes of sensory information – in our case study, whether an actor is reaching a small or a big object. This can be formulated in terms of saliency maps that encode the information gain (or epistemic value) of sampling the next location in the visual field. In turn, this depends upon predictions about the likely configuration of the world based upon a forward or generative model of unfolding events (i.e., the prediction of the hand movement and shape, depending on the actor's goal of grasping a small or a big object). This construction is both principled and straightforward: it differs fundamentally from previous treatments of salience, because salience becomes an explicit function of beliefs and predictions about the future and can be constructed on line in a Bayes-optimal fashion. Furthermore, our work provides a formal perspective on mirror neuron-like activity and the key role of active vision in coupling perception and action. This paper presents the basic ideas and establishes their construct validity by showing that one can reproduce (with remarkable accuracy) key phenomena observed in empirical studies of eye movement dynamics during action observation. The ability to model, in formal terms, action observation may have important implications for the modelling of both eye movements and their neuronal correlates.
